# Impacts of climate change and human activities on different degraded grassland based on NDVI

**DOI:** 10.1038/s41598-022-19943-6

**Published:** 2022-09-23

**Authors:** Qingqing Hou, Zhenxia Ji, Hang Yang, Xiaojun Yu

**Affiliations:** 1 College of Grassland Science, Gansu Agricultural University/Key Laboratory of Grassland Ecosystem (Gansu Agricultural University), Ministry of Education/Sino-U.S. Center for Grassland Ecosystem Sustainability/Pratacultural Engineering Laboratory of Gansu Province, Lanzhou, 730070 China; 2 College of Natural Resources and Environment, Northwest A&F University, Yangling, 712100 China

**Keywords:** Ecosystem ecology, Grassland ecology, Macroecology

## Abstract

Grassland degradation has emerged as a serious socio-economic and ecological problem, endangering both long-term usage and the regional biogeochemical cycle. Climate change and human activities are the two leading factors leading to grassland degradation. However, it is unclear what the degradation level caused by these two factors is. Using the normalized difference vegetation index (NDVI) and coefficient of variation of NDVI (CV_NDVI_), the spatial distribution features of grassland degradation or restoration were analyzed in Qilian County in the northeast of the Qinghai–Tibet Plateau. The dominant climate variables affecting NDVI variation were selected through the combination of random forest model and stepwise regression method to improve the residual trend analysis, and on this basis, twelve possible scenarios were established to evaluate the driving factors of different degraded grasslands. Finally, used the Hurst index to forecast the trend of grassland degradation or restoration. The results showed that approximately 55.0% of the grassland had been degraded between 2000 and 2019, and the area of slight degradation (NDVI_slope_ > 0; CV_NDVI (slope)_ > 0; NDVI_value_ > 0.2) accounted for 48.6%. These regions were centered in the northwest of Qilian County. Climate and human activities had a joint impact on grassland restoration or degradation. Human activities played a leading role in grassland restoration, while climate change was primarily a driver of grassland degradation. The regions with slight degradation or re-growing (NDVI_slope_ > 0; CV_NDVI (slope)_ > 0), moderate degradation (NDVI_slope_ < 0; CV_NDVI (slope)_ > 0), and severe degradation or desertification (NDVI_slope_ < 0; CV_NDVI (slope)_ < 0) were dominated by the joint effects of climate and anthropogenic activity accounted for 34.3%, 3.3%, and 1.3%, respectively, of the total grassland area. Grasslands in most areas of Qilian County are forecasted to continue to degrade, including the previously degraded areas, with continuous degradation areas accounting for 54.78%. Accurately identifying the driving factors of different degraded grassland and predicting the dynamic change trend of grassland in the future is the key to understand the mechanism of grassland degradation and prevent grassland degradation. The findings offer a reference for accurately identifying the driving forces in grassland degradation, as well as providing a scientific basis for the policy-making of grassland ecological management.

## Introduction

Grasslands account for approximately 20% of global land area. Grasslands are important as the production base of material products, but also because of their function to protect biodiversity and provide ecosystem services. Grasslands play an essential role in regional ecological security and socio-economic development^1–3^. However, as extreme weather events coupled with negative anthropogenic activities have risen over the past decades, ecological problems such as grassland degradation have severely increased in many regions globally^3,4^. Nearly 39.06% of global grassland area experienced degradation between 2000 and 2019^3^. In China, degraded grassland accounted for 22.7% of total grassland area between 1982 and 2010^2^. Approximately 38.8% of the grasslands on the Qinghai–Tibet Plateau of China also suffered degradation between 2001 and 2013^5^. This indicates that grassland degradation has become a serious environmental and socio-economic problem, which will gradually threaten the sustainable utilization of grassland resources and the regional biogeochemical cycle^4,5^.

Climate change and human activities are the two leading factors driving terrestrial ecosystem change^6,7^. Generally, they separately or together affected grassland changes on a regional level^8^. Zhou et al. and He et al. distinguished the effects of climate and human factors on grassland degradation. The former determined that the contributions of the two factors to grassland degradation in China were almost at an equilibrium from 1982 to 2010. He et al. demonstrated that the grassland degradation in the Liao River Basin from 1999 to 2009 was driven by both natural processes and human activities^2,6^. In particular, climate change, especially warming, extreme weather phenomena, and altered precipitation patterns will affect the vegetation growing season length, physiological processes, primary productivity, and biodiversity, ultimately impacting the growth and causing degradation of grasslands^9^. In addition, human activities are also the key factors affecting grassland degradation. Among them, overgrazing is regarded as the leading human cause^10^. Overgrazing can lead to a decrease in primary productivity of vegetation and changes in soil characteristics^2^. Therefore, identifying and evaluating the impacts of climate change and human activities on grassland ecosystems is important and may facilitate the development of restoration mechanisms to prevent further grassland degradation^1,6^.

Grasslands degradation monitoring has typically been conducted by field surveys and field control experiments, which can determine the degradation levels and contributing factors^11^. However, this method primarily focuses on small-scale and short-term research and it is inefficient in some areas where field investigations are difficult to conduct^12^, whereas grassland degradation is a protracted and progressive process. As a result, studying grassland degradation on a larger and long-term scale may present some limitations^13^. Remote sensing data can be obtained for larger-scale and longer-term evaluation and can reveal the historical dynamic change law of grassland dynamics. Moreover, the grassland in our study area covered a large spatial region and remote sensing methods are more effective at monitoring grassland degradation^14^, for example, the normalized difference vegetation index (NDVI) and net primary productivity (NPP)^15,16^. The influence of climate change and human activities on grassland changes at the regional^17–19^, watershed^20^ and national scales^9,21^ have recently been studied using remote sensing technology. However, there is no consensus on the primary causes and their different impacts on grassland dynamics^1^.

Traditional quantitative evaluation methods such as multivariate analysis and principal component analysis simply establish the statistical relationship between grassland change and driving factors, but these methods rely primarily on statistical models and ignore the true ecological significance of grassland change^9,22^. Evans and Geerken proposed a residual trend analysis (RESTREND) based on NDVI providing a new method to distinguish the impact of climate change and human activities on grassland change^23^. This method assumes that climate change is the only driving factor, establishes the regression equation between climate change and NDVI, calculates the predicted NDVI, and uses the residual between the actual value and predicted NDVI as the anthropogenic impact to identify the main factors affecting grassland degradation^6^. The RESTREND method has since been frequently utilized to assess the influence of climate and human factors on grasslands on a regional scale and the spatial heterogeneity of the driving factors influencing grassland degradation on a pixel scale^24–26^. Meng et al. used RESTREND and showed that the growth of grasslands and shrublands in the Mongolian Plateau was predominantly affected by human activities^24^, while He et al. found that natural processes and human activities both contributed to grassland degradation in the Liao River Basin^6^. Arden et al. proposed using time series segmentation and RESTREND to detect dryland degradation^25^, and Melakeneh et al. applied this method to assess the rangeland degradation in New Mexico^26^. However, when this methodology was used to fit the multiple regression equation between climate elements and NDVI, there is no clear approach on how to select climate elements.

The previous studies are confined to separate the influence of climate change and human activities on grassland degradation or restoration at the regional scale, but did not account for the differences in the response to these impacts of grassland with different degradation levels on a large scale. Different degraded grasslands have different vegetation and soil characteristics, such as species richness, soil water holding capacity, water use strategies, and soil organic carbon^27^, coupled with the high spatial heterogeneity in the impact of climate change on ecosystem dynamics, particularly in fragile ecosystems at higher altitudes^28,29^. Therefore, different degraded grasslands respond differently to climate change. Song et al. studied the influence of temperature change on nitrogen mineralization in alpine wetlands soils with varying degradation gradients^30^. Additionally, Dai et al. proposed that grazing management had varied effects on alpine grassland at different stages of degradation^31^. However, the degree of grassland degradation caused by climate change and human activities at the regional scale remains uncertain. In addition, researchers also study and predict future changes in grasslands, so that management plans can be prepared in advance. Liu et al. superimposed the Hurst index with the changing trend of NPP and identified the regions in China (e.g., Gannan and Ningxia) with a consistent decrease in grassland NPP, accounting for 17.59% of the overall grassland area^32^. This approach has been used to determine the dynamic change of grassland in the forecasted future based on the superimposed results of the overall change in NDVI and Hurst exponent of NDVI in Central Asia. Areas with continuous improvement and those with continuous degradation accounted for 51.02% and 30.15%, respectively, of the total vegetated area^8^.

Vegetation fragmentation is a common feature of arid-ecosystem degradation, that lead to the presence of bare-soil patches and results in changes in vegetation cover and spatial heterogeneity^33,34^. The ecosystems of the Qinghai–Tibet Plateau are fragile and sensitive to climate change and human activities^28^, and the grassland ecosystem has been degraded^4^. In addition, the natural grassland has been encroached by a large number of invasive species, and the bare-soil patches eventually turned into large areas of “black soil beach,” resulting in the spatial heterogeneity that characterizes other arid ecosystems^34^. The Qilian County is located in the northeast of Qinghai- Tibet Plateau. The grassland covers most of the area, and it is very vulnerable to the influence of extreme weather conditions. There are few studies on the law of grassland dynamic change and its dominant factors, as well as the grassland degradation or restoration.

Therefore, this study selects the NDVI and its coefficient of variation (CV_NDVI_) to quantify the spatial heterogeneity and evaluate grassland degradation. Using Qilian County as the study region, the geographical distribution of grassland degradation or restoration is determined by analyzing the changing trend in NDVI and CV_NDVI_ during a long-term time series (i.e., between 2000 and 2019). Combined with the random forest model and stepwise regression method, the dominant climate variables affecting NDVI change are selected, and the regression equation between NDVI and climate elements is established to improve the residual trend analysis. According to the relative effects of climate change and human activities on the NDVI and CV_NDVI_, twelve possible scenarios are established to redefine the driving factors of grassland degradation. Finally, the Hurst index was used to forecast the change of grassland degradation or restoration. The findings of the study can provide new insights for separating the influence of climate change and human activities on different degraded grasslands and in clarifying the degradation degrees caused by climate change and human activities.

## Materials and methods

### Study area

**Figure 1:**
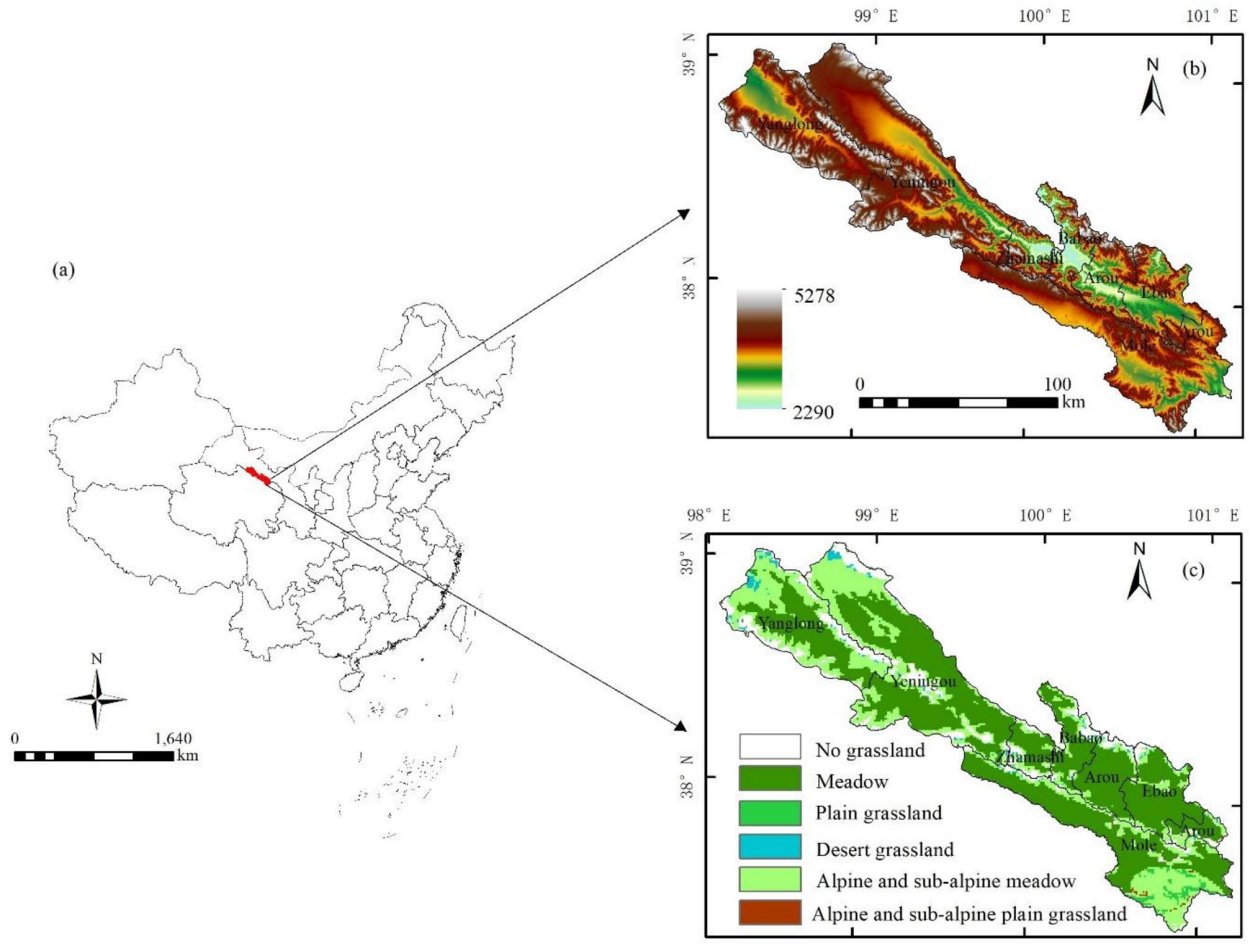
Geographical location (**a**), altitude (**b**), and the distribution of grassland types (**c**) in Qilian County. The map was generated by ArcGIS 10.2, URL: https://www.esri.com.

The Qilian County is located in the northeast of the Qinghai–Tibet Plateau and the middle part of Qilian Mountain (Fig. 1a). The mountainous terrain of Qilian County is complex and the vegetation cover types are diverse. The average altitude is 3169 m (Fig. 1b). The vertical changes of climate, soil and vegetation are obvious, and the temperature decreases with the gradual increase of altitude. The annual average temperature is—1.8– 1.0 °C, and the annual precipitation of 406.7 mm^35^. Total grassland area is 1.12 × 10^6^hm^2^, accounting for 75.74% of the total area of the county. The grassland types mainly include Meadow, Plain grassland, Desert grassland, Alpine and sub-alpine meadow, and Alpine and sub-alpine grassland (Fig. 1c).

### Data source and preprocessing

The NDVI data of Qilian Mountain for 2000–2019 were obtained from the National Cryosphere Desert Data Center (http://www.crensed.ac.cn/portal/metadata/a9f71fb8-1b0a-4607-9526-88930d04d134) of China, with a spatial resolution of 30 m. The NDVI of the study area was obtained after clipping. The land cover data were acquired from the Global Land Cover 2000 (GLC2000) product (https://forobs.jrc.ec.europa.eu/products/glc2000/products.php), with a spatial resolution of 1 km, which was utilized to obtain the grassland cover data. Road and river network data from OpenStreetMap (http://download.geofabrik.de/asia/china.html) were used to conceal greater spatial heterogeneity values generated by roads and rivers. The monthly meteorological data used herein included temperature, precipitation, sunshine hours, wind speed, and solar radiation. The data of temperature, precipitation, sunshine hours, and wind speed were obtained from the National Earth System Science Data Center (http://www.geodata.cn/data/index.html?publisherGuid=126744287495931&categoryId=4) with a spatial resolution of 1 km. The solar radiation data were from the National Science & Technology Infrastructure (http://data.tpdc.ac.cn/zh-hans/), with a spatial resolution of 10 km. All meteorological data were processed and resampled to obtain annual scale data with 30 m resolution, which was used to judge the dominant climate factors affecting the NDVI.

### Data analysis

#### NDVI coefficient of variation (CV_NDVI_)

The CV_NDVI_ is a measurement of the relative fluctuation stability of the change in a variable^32^. To assess the spatial heterogeneity of vegetation cover, the CV_NDVI_ inside a 3 × 3-pixel moving window of 90 × 90 m area was determined. River and road networks were removed from the computation of the CV_NDVI_ since the substantial spatial heterogeneity surrounding them may not imply degradation as assumed^13^. In this paper, the CV_NDVI_ was employed to examine the stability of changes in grassland NDVI over a long period, computed as follows:

1 $${CV}_{NDVI}=\frac{\sqrt{\frac{\sum_{i=1}^{n}({NDVI}_{i}-\overline{NDVI })}{n-1}}}{\overline{NDVI} }$$

where ${CV}_{NDVI}$ is the coefficient of variation of NDVI; *n* is the duration of the study; ${NDVI}_{i}$ indicates NDVI in year $i$, and $\overline{NDVI }$ is the average NDVI value during the study period.

#### Trend analysis

Based on the pixel scale, the Sen’s slope estimation method^36^ was used to estimate the spatial trend of NDVI and CV_NDVI_ variation between 2000 and 2019 in Qilian County. The Mann Kendall statistical test (M–K) was used for the significance of the changes^37,38^. The Sen’s slope in Eq. (2), and the M–K formulae are shown in Eqs. (3)– (5).

2 $$M= Median \left(\frac{{a_{j} - a_{i} }}{j - i} \right),\forall j > i$$

where *M* is the slope, Median is the median function, and *i* and *j* represent the year. If M > 0, it shows a positive trend of the NDVI or CV_NDVI_ in the pixel; if M < 0, it means that the NDVI or CV_NDVI_ in the pixel has a negative trend.

3 $${Z}_{c}=\left\{\begin{array}{ll}\frac{S-1}{\sqrt{Var\left(S\right)}}, & \quad S>0\\ 0, & \quad S=0\\ \frac{S+1}{\sqrt{Var(S)}} , & \quad S<0\end{array}\right.$$

4 $$S=\sum_{i=1}^{n-1}\sum_{j=j+1}^{n}sgn({a}_{j}-{a}_{i})$$

5 $$sgn\left({a}_{j}-{a}_{i}\right)=\left\{\begin{array}{ll}1, &\quad {a}_{j}-{a}_{i}>0\\ 0,&\quad{ a}_{j}-{a}_{i}=0\\ -1,&\quad{ a}_{j}-{a}_{i}<0\end{array}\right.$$

where ${a}_{i}$ and ${a}_{j}$ are the $i$-th and $j$-th data values, respectively, and $n$ is the length of the time series. Z is the statistical value of the M–K test, and $sgn$ is the signum function. For a confidence level $p$, the Z value should satisfy the formula of $\left|{Z}_{c}\right|>{Z}_{1-p/2}$.

#### Grassland degradation levels

The annual median NDVI and CV_NDVI_ inside 3 × 3-pixel moving windows were calculated for each year between 2000 and 2019, and the trend analysis method (2.2.2) was used to study long-term change trends in grassland cover. The slope of NDVI and CV_NDVI_ were used to determine grassland degradation levels, as shown in Table 1. A detailed description of the grassland degradation or restoration levels can be found in Li et al.^13^.

**Table 1:** Degrees of grassland degradation or restoration.

Grassland dynamics	NDVI _slope_	CV_**NDVI** (slope)_	NDVI _value_	Grassland degradation levels
Grassland restoration	> 0	< 0		Improving conditions
	> 0	> 0	NDVI < 0.2	Regrowing conditions
Grassland degradation	> 0	> 0	NDVI > 0.2	Slight degradation
	< 0	> 0		Moderate degradation
	< 0	< 0	NDVI > 0.2	Severe degradation
	< 0	< 0	NDVI < 0.2	Desertification

#### Grassland degradation hot plots

The hot plots of grassland degradation were classified as places where the NDVI was highly concentrated in grassland degradation area. The hot plots were detected using the Anselin Local Moran’s indicator clustering approach, an index frequently used to analyze the spatial clustering properties of a variable^36,39^.

#### Improved residuals analysis

The residual analysis method can discriminate between the impact of climate change and human activities on ecological parameters^23^. A regression model of climate parameters and the annual maximum NDVI values is first created using multiple correlation regression. This connection may then be used to calculate the predicted NDVI. However, when establishing the multiple regression equation of the NDVI and climate elements, most studies directly use precipitation and temperature as independent variables^24^. Therefore, to improve the fitting degree of the regression equation, the random forest regression model and stepwise regression method were combined to select the dominant climate variables affecting the NDVI variations. The random forest regression model can rank the importance of multiple independent variables according to dependent variables, with larger values indicating the greater importance^40^. The stepwise regression method is effective for analyzing multiple variables in multiple linear regression analysis. By introducing independent variables one by one, the variables with significant influence are retained, and the variables with insignificant influence are eliminated. Finally, the fitting degree of the regression equation tends to be perfect^41,42^.

According to the results shown in Fig. S1 and Table S1, temperature, precipitation, and sunshine hours were finally selected as the climatic factors for predicting the NDVI. Next, a three-element linear regression equation for the annual NDVI and climatic factors was established pixel by pixel between 2000 and 2019. The NDVI affected by climate on the pixel scale of Qilian County for this period can be obtained from the following regression model:

6 $${NDVI}_{C}=aT+bP+cS+d$$

where ${NDVI}_{C}$ is the predicted NDVI; *a*, *b*, and *c* are the regression coefficients of T, P, and S, respectively; T, P and S are the annual average temperature (℃), annual accumulated precipitation (mm), and annual accumulated sunshine hours (h), respectively; *d* is a constant.

The NDVI residuals were calculated as the differences between the predicted and observed NDVI values, which can represent the impact of human activities on the NDVI^23^. To distinguish the CV_NDVI_ change caused by climate variation from that caused by human activities, the CV of the predicted NDVI was calculated to obtain the predicted CV_NDVI_, and then the CV_NDVI_ residuals.

7 $${NDVI}_{H}={NDVI}_{obs}-{NDVI}_{C}$$

8 $${CV}_{NDVI(H)}={CV}_{NDVI(obs)}-{CV}_{NDVI(C)}$$

where ${NDVI}_{H} \;\;or \;\;{CV}_{NDVI(H)}$ is the NDVI or CV_NDVI_ residuals; ${NDVI}_{obs} \;\;or \;\;{ CV}_{NDVI(obs)}$ is the observed NDVI or CV_NDVI_ values based on remote sensing images.

#### Grassland degradation scenario analysis and quantitative assessment method

The trend of ${NDVI}_{C}$, ${CV}_{NDVI(C)}$, ${NDVI}_{H}$, and ${CV}_{NDVI(H)}$ between 2000 and 2019 were calculated, which represented the change trends in NDVI and CV_NDVI_ under the influence of climate variation and human activities. If the trend is positive, climate change or human activities could contribute to an increase in NDVI or spatial heterogeneity. Conversely, it will lead to a decrease in NDVI or spatial heterogeneity. The classification of the main driving factors of NDVI or CV_NDVI_ change are listed in Table. 2, with the calculated relative contribution rate of climate change and human activities to NDVI or CV_NDVI_ change.

**Table 2:** Classification criterion and contribution rate of driving factors of NDVI or CV_NDVI_.

Definition of driving factors	Classification criterion of driving factors			The contribution rate of driving factors (%)	
	Slope (${NDVI}_{obs}$) or Slope (${CV}_{NDVI(obs)}$)	Slope (${NDVI}_{C}$) or Slope (${CV}_{NDVI(C)}$)	Slope (${NDVI}_{H}$) or Slope (${CV}_{NDVI(H)}$)	Climate variation	Human activities
Climate	> 0	> 0	< 0	100	0
Human	> 0	< 0	> 0	0	100
Climate and human	> 0	> 0	> 0	$\frac{slope\left({NDVI}_{C}\right) \;\; or\;\; slope \;\;({CV}_{NDVI(C)})}{slope\left({NDVI}_{obs}\right) \;\;or \;\;slope({CV}_{NDVI(obs)})}$	$\frac{slope\left({NDVI}_{H}\right) \;\;or\;\; slope\;\;({CV}_{NDVI(H)})}{slope\left({NDVI}_{obs}\right) \;\;or \;\;slope({CV}_{NDVI(obs)})}$
Climate	< 0	< 0	> 0	100	0
Human	< 0	> 0	< 0	0	100
Climate and human	< 0	< 0	< 0	$\frac{slope\left({NDVI}_{C}\right) \;\; or\;\; slope ({CV}_{NDVI(C)})}{slope\left({NDVI}_{obs}\right) \;\; or \;\;slope({CV}_{NDVI(obs)})}$	$\frac{slope\left({NDVI}_{H}\right) \;\; or\;\; slope({CV}_{NDVI(H)})}{slope\left({NDVI}_{obs}\right) \;\;or \;\;slope({CV}_{NDVI(obs)})}$

The driving factors of grassland degradation or restoration were redefined (Table 3) based on the respective roles of climate and human activities on NDVI and CV_NDVI_, to develop twelve scenarios (scenarios 1‒12). There are three scenarios (scenarios 1‒3) for each restored grassland pixel: climate-driven grassland improvement (scenario 1, CDI), grassland improvement because of human activity (scenario 2, HDI), and the combined effect of both drivers on grassland improvement (scenario 3, BDI).

**Table 3:** The twelve scenarios for determining the roles of climate and human activities in grassland dynamic.

Grassland status	Scenario	${\mathrm{R}}_{\mathrm{NDVI}}$	${\mathrm{R}}_{\mathrm{CV}}$	Definition of grassland change drivers
Grassland restoration	Scenario1 (CDI)	CDNI	CDCD	Climate-driven grassland improvement
		CDNI	BDCD	
		BDNI	CDCD	
	Scenario2 (HDI)	HDNI	HDCD	Human activities-driven grassland improvement
		HDNI	BDCD	
		BDNI	HDCD	
	Scenario3 (BDI)	CDNI	HDCD	Both of the two drivers effected grassland improvement
		HDNI	CDCD	
		BDNI	BDCD	
Grassland degradation	Scenario4 (CDSR)	CDNI	CDCI	Climate- driven grassland with slight degradation or re-growing
		CDNI	BDCI	
		BDNI	CDCI	
	Scenario5 (HDSR)	HDNI	HDCI	Human activities-driven grassland with slight degradation or re-growing
		HDNI	BDCI	
		BDNI	HDCI	
	Scenario6 (BDSR)	CDNI	HDCI	Both of the two drivers effected grassland with slight degradation or re-growing
		HDNI	CDCI	
		BDNI	BDCI	
	Scenario7 (CDMD)	CDND	CDCI	Climate-driven grassland with moderate degradation
		CDND	BDCI	
		BDND	CDCI	
	Scenario8 (HDMD)	HDND	HDCI	Human activities-driven grassland with moderate degradation
		HDND	BDCI	
		BDND	HDCI	
	Scenario9 (BDMD)	CDND	HDCI	Both of the two drivers effected grassland with moderate degradation
		HDND	CDCI	
		BDND	BDCI	
	Scenario10 (CDSD)	CDND	CDCD	Climate-driven grassland with severe degradation or desertification
		CDND	BDCD	
		BDND	CDCD	
	Scenario11 (HDSD)	HDND	HDCD	Human activities-driven grassland with severe degradation or desertification
		HDND	BDCD	
		BDND	HDCD	
	Scenario12 (BDSD)	CDND	HDCD	Both of the two drivers effected grassland with severe degradation or desertification
		HDND	CDCD	
		BDND	BDCD	

${R}_{NDVI}$: The relative roles of climate and human activities on NDVI; ${R}_{CV}$: The relative roles of climate and human activities on CV_NDVI_; CDNI: Climate-driven NDVI increase; BDNI: Both of the two drivers effected NDVI increase; HDNI: Human activities-driven NDVI increase; CDND: Climate-driven NDVI decrease; BDND: Both of the two drivers effected NDVI decrease; HDND: Human activities-driven NDVI decrease; CDCI: Climate-driven CV_NDVI_ increase; BDCI: Both of the two drivers effected CV_NDVI_ increase; HDCI: Human activities-driven CV_NDVI_ increase; CDCD: Climate-driven CV_NDVI_ decrease; BDCD: Both of the two drivers effected CV_NDVI_ decrease; HDCD: Human activities-driven CV_NDVI_ decrease.

There are nine scenarios (scenario 4‒12) for each degraded grassland pixel: climate-driven grassland with slight degradation or re-growing (scenario 4, CDSR), grassland with slight degradation or re-growing because of human activity (scenario 5, HDSR), effect of both drivers on grassland with slight degradation or re-growing (scenario 6, BDSR), climate-driven grassland with moderate degradation (scenario 7, CDMD), grassland moderately degraded by human activities (scenario 8, HDMD), effect of both drivers on moderately degraded grassland (scenario 9, BDMD), grassland with severe degradation or desertification caused by climate change (scenario 10, CDSD), grassland with severe degradation or desertification as a result of human activity (scenario 11, HDSD), and effect of both drivers on grassland with severe degradation or desertification (scenario 12, BDSD).

#### Hurst exponent (H)

Since the Hurst exponent can effectively depict self-similarity and long-term reliance, it is commonly employed in climatology and vegetation studies to analyze the durability of long-term changes in time series data^43,44^. In this study, the Hurst exponent was estimated by R/S analysis, and the change characteristics of NDVI and CV_NDVI_ were analyzed pixel by pixel to reflect the persistence of the changing trend as follows:

1. The time series is *A* [${A}_{i}$] (*i* = 1,2, 3,., *n*), and for each positive integer *m* ≥ 1:
  9 $$\overline{{A }_{i}}=\frac{1}{m}\sum_{i=1}^{m}{A}_{i} i=\mathrm{1,2},\dots ,n$$
2. Cumulative deviation
  10 $${X}_{(i,m)}=\sum_{i=1}^{i}({A}_{i}-\overline{{A }_{m}}) 1\le i\le m$$
3. Range
  11 $${R}_{(i)}=max1\le i\le m{X}_{\left(i,m\right)}-min1\le i\le m{X}_{(i,m)}$$
4. Standard deviation
  12 $${S}_{m}={\left[\frac{1}{m}\sum_{i=1}^{m}({A}_{\left(i\right)})-{A}_{m}^{2}\right]}^{1/2}$$
5. Calculating the Hurst exponent index
  13 $$\frac{{R}_{i}}{{S}_{m}}=(c\tau )$$
6. Calculating the H value:
  14 $$\mathrm{log}\left(R/S\right)n=a+H\times \mathrm{log}(n)$$

The Hurst exponent has a distribution range of 0 to 1; if 0.5 < H < 1, the future trend will be consistent with the previous one; if H = 0.5, the future trends cannot be forecast; If 0 < H < 0.5, it implies that the changing trend of the variable will be incompatible with the prior one^32^.

The Hurst exponent of NDVI and CV_NDVI_ was respectively superimposed with their dynamic trend to obtain the corresponding future dynamic trends, and the degradation levels were divided and compared with the degradation levels of 2019 to obtain the forecasted future dynamic change trend of Qilian County.

## Results

### Dynamic variations in the NDVI and CV_NDVI_ between 2000 and 2019

**Figure 2:**
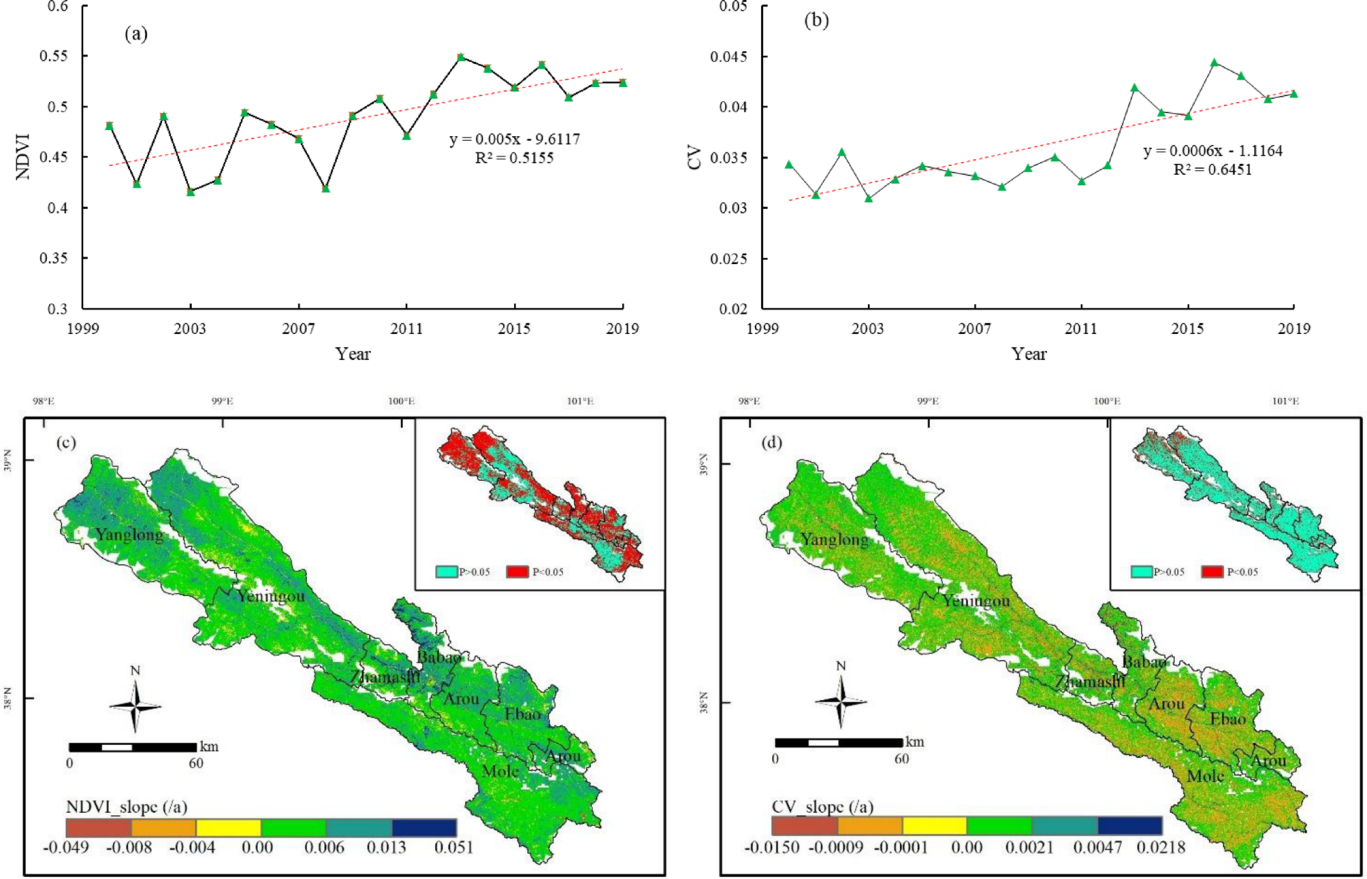
(**a**) and (**b**) Temporal variation in normalized difference vegetation index (NDVI) and coefficient of variation of NDVI (CV_NDVI_) between 2000 and 2019. (**c**) and (**d**) Spatial variation in normalized difference vegetation index (NDVI) and coefficient of variation of NDVI (CV_NDVI_) between 2000 and 2019. The map was generated by ArcGIS 10.2, URL: https://www.esri.com.

As shown in Fig. 2a, the mean annual NDVI of grasslands in Qilian County is 0.49, with the highest and lowest NDVI in 2013 (0.55), and 2008 (0.42), respectively, increasing at a rate of 0.005 between 2000 and 2019 (R^2^ = 0.52). In terms of spatial distribution, the total area with a rising tendency in grassland NDVI is 11.47 × 10^3^ km^2^, accounting for 93.6% of the total grassland area, with the increasing trend being most visible in Babao Town, Zhamashi Township, and the northwestern part of Yanglong Township. The area with a downward trend in NDVI accounts for 6.4%, located around the northwest of Yeniugou Township and along roads and rivers. The changing trend in grassland NDVI accounted for 58% of the areas passing the significance test (Fig. 2c).

Regarding the CV_NDVI_ (Fig. 2b), it also shows an uptrend between 2000 and 2019 (slope = 0.0006; R^2^ = 0.65). The area CV_NDVI_ with an increasing trend is 7.10 × 10^3^ km^2^, accounting for 58.0% of the total grassland area, which is mainly distributed in the northwest of Yeniugou Township and Yanglong Township, indicating that the stability of grassland patches in these areas is reduced. Moreover, 42.0% of the grassland area shows a decreasing trend in grassland CV_NDVI_, especially around Arou Township and Ebao town in the southeast of Qilian County. In the variation trend of grassland CV_NDVI_, 20.2% can pass the significance test, which is mainly located in the northwest of Qilian County (Fig. 2d).

### Spatial distribution of grassland degradation and degradation hot plots

**Figure 3:**
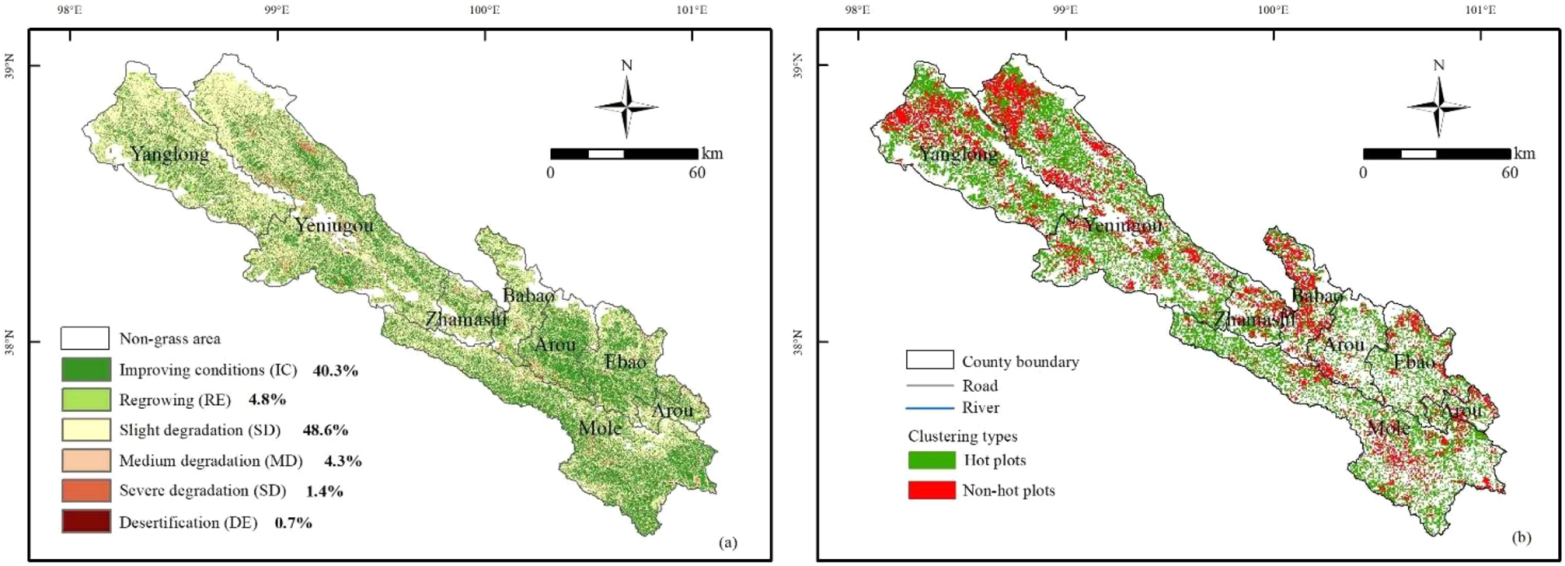
Spatial variation of grassland degradation or restoration for the 2000–2019 period. The map was generated by ArcGIS 10.2, URL: https://www.esri.com.

The spatial distribution of grassland degradation or restoration in Qilian County between 2000 and 2019 was obtained from the trend of NDVI and CV_NDVI_ (Fig. 3a). Approximately 55.0% of the grassland of Qilian County has been degraded, of which the area of slight degradation accounts for 48.6% of the total grassland area. These regions are concentrated in Qilian County’s northwest. The moderate degradation is largely scattered around Yeniugou Township, Babao Town, and Arou Township, which occupies 4.3% of the grassland. Only 1.4% of the whole region has suffered severe degradation, with almost no desertification (0.7%). On the contrary, the restored grasslands area accounts for 45.1% of the total grassland area, and the regions with improving conditions are mainly concentrated in the southeast of Qilian County, such as Arou Township, Ebao Town, and Mole Town, accounting for 40.3%. In conclusion, the spatial distribution pattern of grassland degradation or restoration in Qilian County demonstrated great spatial heterogeneity, with less restoration area than the degradation area (45.1% < 55%); while the southeast was dominated by restoration, the northwest was dominated by degradation.

The results of the cluster analysis further reveal that the degraded hot plots are mostly in a point distribution pattern, with no significant clustering characteristics (Fig. 3b). The statistics indicate that 33.7% of grassland degradation had no clustering characteristics, mainly scattered in the northwest of Yanglong Township and Yeniugou Township, and some regions of Babao Town. Approximately 66.3% of grassland degradation was clustered and densely distributed in the northwest of Qilian County.

### The impact of climate change and human activities on grasslands dynamics

#### The impact of climate change on NDVI and CV_NDVI_

**Figure 4:**
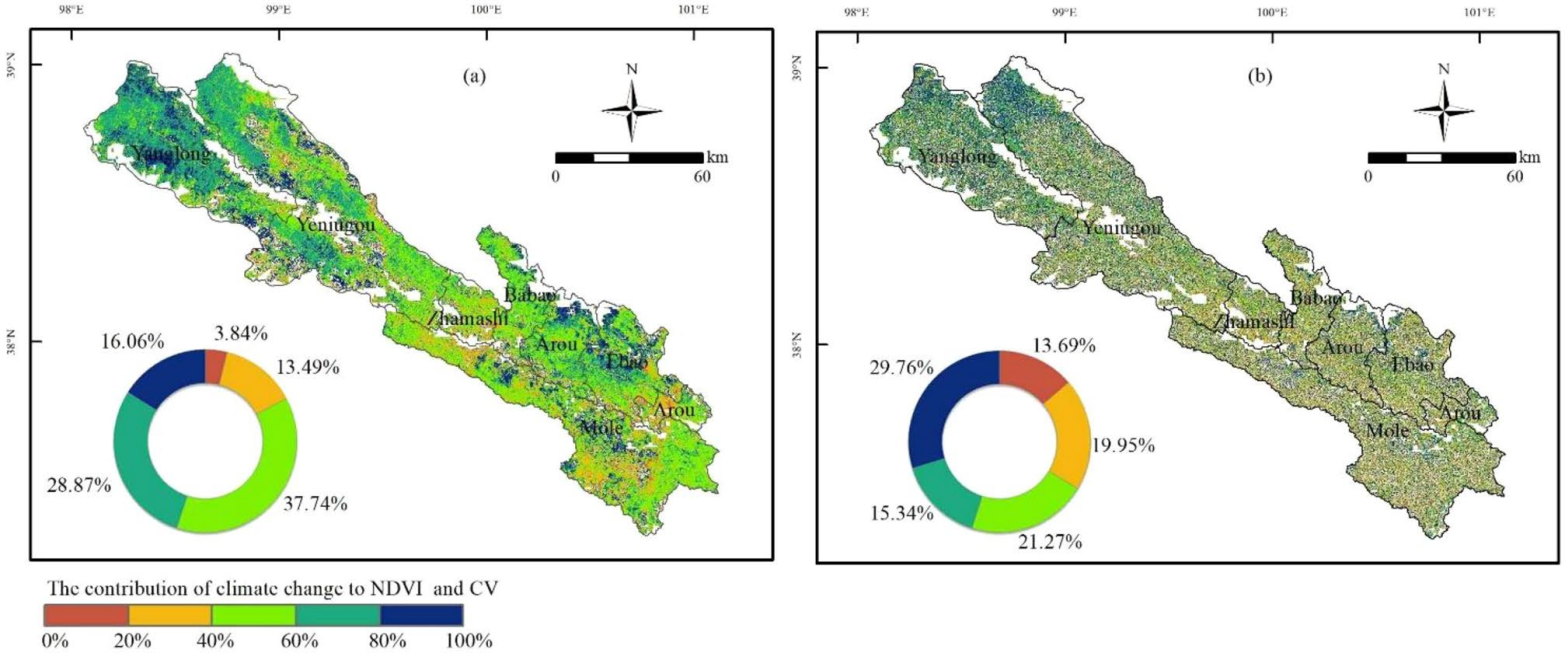
Contribution of climate change to the NDVI (**a**) and CV_NDVI_ (**b**) in Qilian County between 2000 and 2019. The map was generated by ArcGIS 10.2, URL: https://www.esri.com.

As shown in Fig. 4, there is large spatial heterogeneity in the impact of climate variation on NDVI and CV_NDVI_ dynamic changes in Qilian County between 2000 and 2019 (Fig. 4). The contribution of climate variation to NDVI is mostly above 40%, and the area with a contribution of 40–60% accounts for 37.74% of the total grassland area. Most areas of Yanglong Township and some of Yeniugou Township in the northwest of Qilian County contribute more than 60% to climate variation, indicating that the northwest has been more vulnerable to climate change (Fig. 4a). The contribution of climate variation to CV_NDVI_ is similar to that of NDVI in spatial distribution. Overall, the contribution of climate variation in the northwest is higher than that in the southeast. The area with 80–100% contribution accounts for 29.76% of the total grassland area, mostly around Yanglong Township and Yeniugou Township in the northwest, while Ebao Town, Mole Town, and Arou Township in the southeast contributed less than 40%, indicating that CV_NDVI_ affected by human activities in the southeast is above 60% (Fig. 4b).

#### Effects of climate and human activities on grassland dynamic

**Figure 5:**
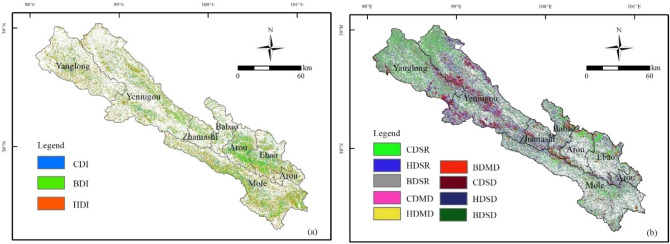
Effects of climate and human activities on grassland degradation (**b**) and restoration (**a**). The map was generated by ArcGIS 10.2, URL: https://www.esri.com.

**Figure 6:**
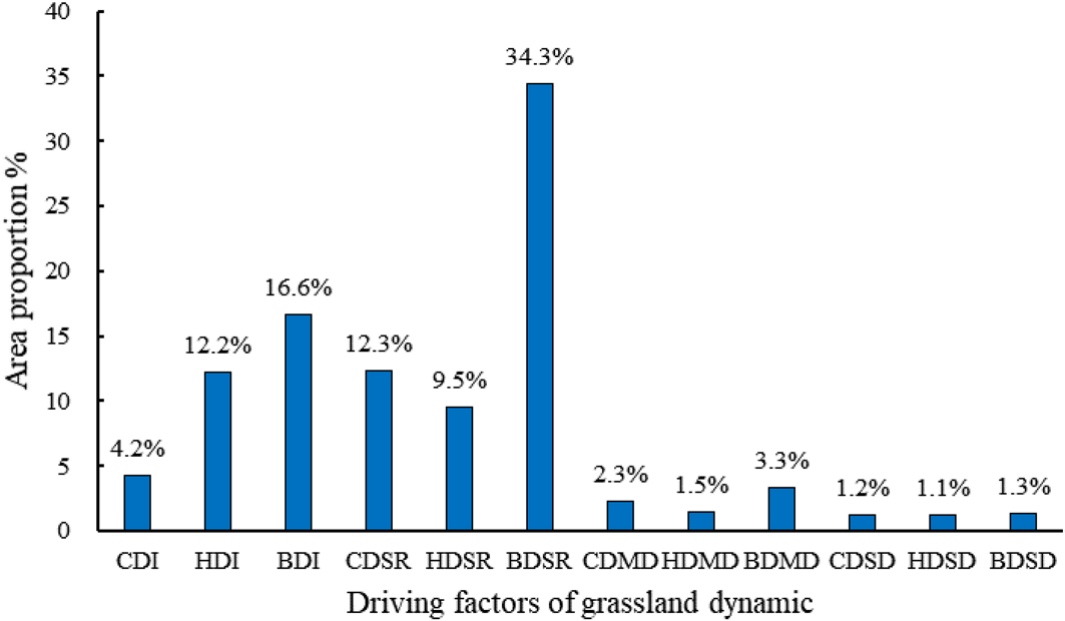
Area proportion of driving factors for grassland degradation and restoration.

Based on the twelve grassland dynamic change scenarios, the spatial distribution of grassland degradation or restoration caused by climate change, human activities, and their joint effect between 2000 and 2019 was determined (Fig. 5a). The grassland improvement regions (BDI) induced by the two factors account for 16.6%, mainly located around Arou Township and Ebao Town in the southeast of Qilian County. The human activities (HDI) and climate-driven (CDI) grassland improvement regions account for 12.2% and 4.2%, respectively, which were scattered across Qilian County. Spatially, the regions of climate-driven grassland with slight degradation or re-growing (CDSR) were observed around Yanglong Township, the northwest of Yeniugou Township, and the north of Arou Town, accounting for 12.3% (Figs. 5b, 6). The regions where human activities-driven grassland with slight degradation or re-growing (HDSR) account for 9.5%, with scattered distribution (Figs. 5b, 6). The regions of grassland with slight degradation or re-growing were dominated by both drivers (BDSR) account for the largest proportion, reaching 34.3%, and are mainly located around Zhamashi Township and Babao Town in the middle of Qilian County, as well as the northwest of Yanglong Township and Yeniugou Township. Furthermore, the grassland with moderate degradation caused by climate change (CDMD), human activities (HDMD), and both drivers (BDMD) are mainly concentrated in the northwest of Qilian County (Yeniugou Township, the border region between Yeniugou Township and Yanglong Township), accounting for 7.1%. The grassland with severe degradation is mainly controlled by climate change (CDSD), human activities (HDSD), and both drivers (BDSD) account for a relatively modest proportion, that is, 1.2%, 1.1%, and 1.3% respectively, located around Yeniugou Township (Figs. 5b, 6).

In summary, climate change and human activities jointly affected grassland restoration or degradation in Qilian County between 2000 and 2019. The contribution of the CDI region was less than that of the HDI region (4.2% < 12.2%), implying that human activities dominated grassland restoration in Qilian County for the 2000–2019 period. Secondly, regardless of the degree of degradation, the area of climate-driven grassland degradation was larger than that of human activities-driven, such as slight degradation or re-growing (12.3% vs. 9.5%), moderate degradation (2.3% vs. 1.5%), and severe degradation or desertification (1.2% vs. 1.1%), indicating that climate change played a leading role in grassland degradation.

### Forecasted patterns of grassland dynamics

**Figure 7:**
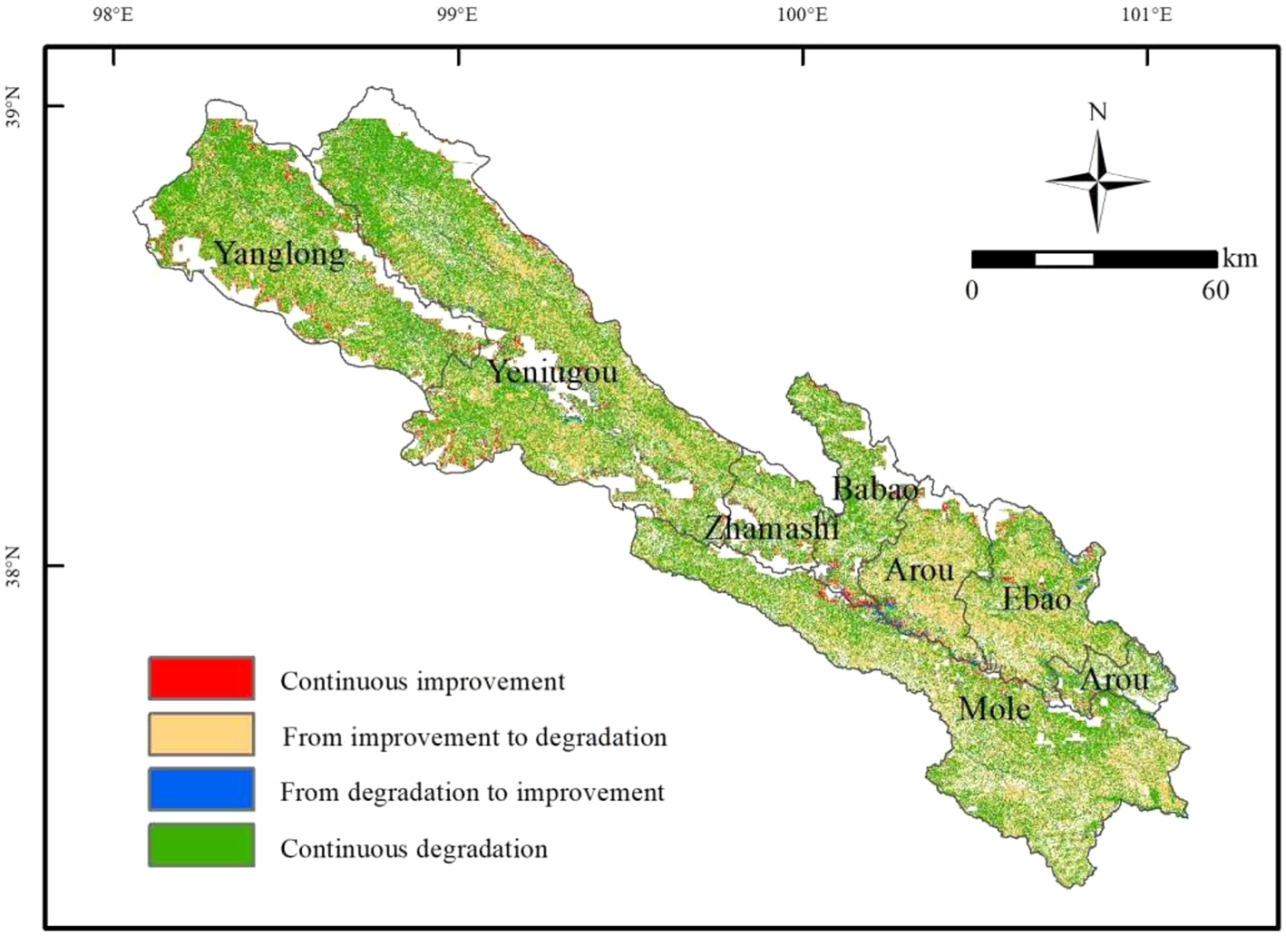
Spatial distribution of the forecasted grassland dynamic. The map was generated by ArcGIS 10.2, URL: https://www.esri.com.

**Figure 8:**
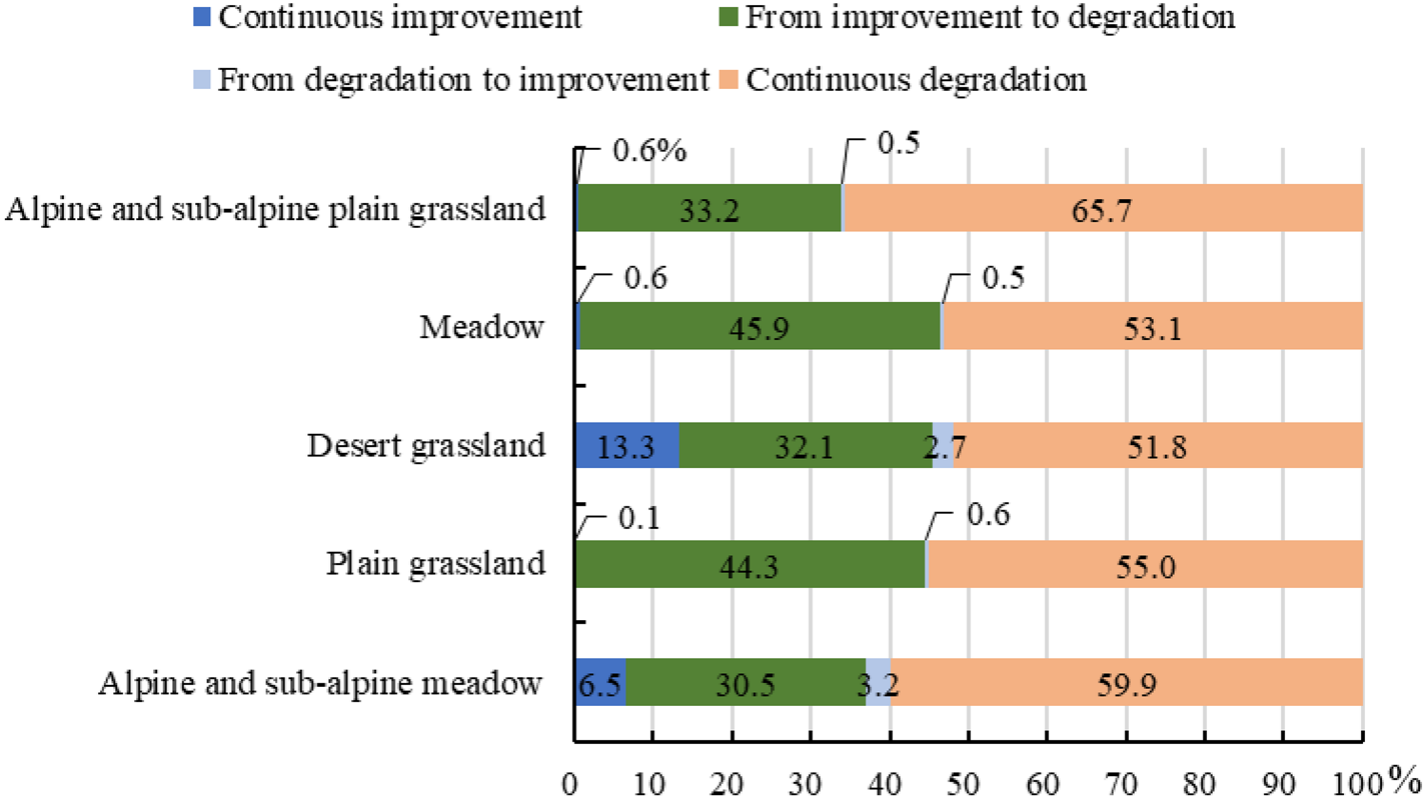
Dynamic changes in grassland types.

The dynamic change trend in grassland in Qilian County shown in Fig. 7 indicate that the area of grassland improvement accounts for only 4.31% of the total grassland area, of which the area of continuous improvement accounts for 2.86%. The area from degradation to improvement accounts for 1.45%, mainly in the south of Babao Town and Arou Town, as well as near the junction of alpine and sub-alpine meadow and bare rocks. Desert grassland and alpine and sub-alpine meadow are the main grassland types (Fig. 8), indicating that the grassland in these areas have been reasonably protected and gradually improved from degradation. The area with grassland degradation accounts for 95.69% of the total grassland, with continuous degradation accounting for 54.78% and the area from improvement to degradation accounting for 40.91%. The region of continuous degradation is mainly distributed in the northwest of Qilian County, especially in the northwest of Yanglong Township and Yeniugou Township. In addition, 65.7% and 59.9% of alpine and sub-alpine plain grassland and alpine and sub-alpine meadows, respectively, have been continuously degraded (Fig. 8). The region from improvement to degradation is mainly Arou Township and Ebao Town. In terms of grassland types, except for the 16.0% and 9.7% of desert grassland and alpine and sub-alpine meadow, respectively, improvement, the other grassland types are degraded. The area of continuous degradation is greater than that from improvement to degradation, which means that most grasslands of Qilian County will continue to degrade, including the previously degraded areas.

## Discussion

### Methodology

The spatial heterogeneity linked to vegetation and soil patches is a valid indicator for assessing degradation in dry environments^45^. Similarly, mixtures of soil and vegetation patches can be found in degraded grasslands on the Qinghai–Tibet Plateau^46,47^. The formation of “black soil patches” during degradation results in the spatially discontinuity of grassland cover, resulting in changes in spatial heterogeneity. The change in grassland cover and spatial heterogeneity was the early warning signal of grassland degradation in the Qinghai–Tibet Plateau^34^. These two indicators can be utilized to determine the degree of grassland degradation. In this study, considering that the vegetation cover in some degraded areas may be higher than that in non-degraded areas such as the higher vegetation cover of invasive species than that of native species^48^, we calculated the coefficient of variation in NDVI within 90 m × 90 m areas consisting of nine 30 m × 30 m pixels defined spatial heterogeneity, which is used to quantify the soil patches in degraded grasslands. In combination with NDVI, the degradation level of Qilian County in 2019 was divided. According to our findings, approximately 55.0% of the grassland in the study area has been degraded, lower area than the 69.05% estimated by Lu et al. for Qilian County^49^. The differences might have occurred because this study used remote sensing data to identify grassland degradation areas from spatial, while the grassland degradation area studied by Lu et al.^49^ was obtained based on statistical data, and over a different research period. In addition, NDVI and NPP showed increasing trends in the Qinghai–Tibet Plateau and most regions of the Qilian Mountains^50–52^, coupled with more volatile precipitation changes, resulting in increasing spatial heterogeneity^39^. This is consistent with the changing trend of NDVI and spatial heterogeneity in Qilian County.

According to the findings of this study, grassland restoration or degradation in Qilian County between 2000 and 2019 was mostly jointly caused by climate change and human activities. The RESTREND method simplifies an otherwise complicated mechanism of grassland degradation or restoration by spatially identifying and quantifying the contribution degree, affected regions, and locations affected by climate change and human activities. It has been frequently used to separate and assess the relative roles of climate change and human activities in grassland degradation on a regional scale^25,26^. Further, this method was also applicable to arid and semiarid regions^35,54^. For example, Li et al. and Jiang et al. used it to differentiate vegetation changes caused by climate and human factors in the Inner Mongolia Xilingol grassland region and Central Asia, respectively^8,53^. Wu et al. examined both the magnitude and direction of climate and human activities influences on NDVI variation over the alpine meadows, steppes, and desert-steppes of the Qinghai–Tibet Plateau^54^. Therefore, we selected the RESTREND method to evaluate the relative contribution of climate change and human activities in grassland degradation in Qilian County. However, when fitting the multiple regression equation between climate variation and NDVI, most studies employed only precipitation and temperature as climate parameters, which may affect the results^24^. Therefore, to select reasonable climate elements and improve the fitting degree of the regression equation, we used stepwise regression and random forest regression model to select the dominant climate elements that have the most significant impact on NDVI variations, and then establish a regression equation with NDVI to predict NDVI. The results showed that when establishing the regression equation between climate elements and NDVI, the accuracy of selecting precipitation, temperature, and sunshine hours was higher than that of selecting only two climate elements of precipitation and temperature, as R^2^ increased by 17% (Table S2). Furthermore, we designed twelve scenarios to distinguish the driving forces of climatic variation and human activities on different degraded grasslands, which may accurately identify the degree of grassland degradation induced by climate change and human activities in a region.

### Impacts of climate change on grassland degradation or restoration

Climate change are changing grassland ecosystems^4^. Temperature and precipitation are the two most important climatic factors influencing the presence and distribution of grassland ecosystems^55^. Therefore, climate change affects the changes in precipitation and temperature by changing and affecting soil moisture, soil microorganisms, photosynthesis, and plant respiration, thereby further controlling grassland growth and ecosystem productivity and affecting degradation or restoration^3^. Climate change was an important driving force affecting grassland degradation in this study. The area of climate-driven grassland degradation was larger than that of human activities-driven, particularly in the northwest of Qilian County. Similar to a previous study^56^, highlighting climate change as the principal driver of nearly half of the global grassland degradation.

**Figure 9:**
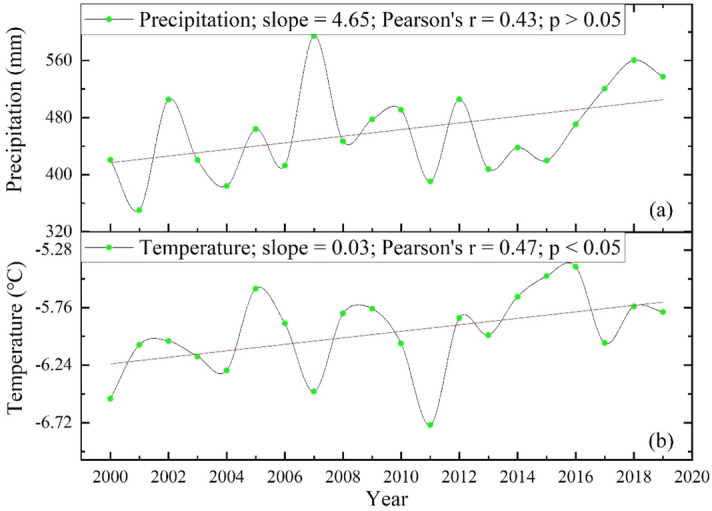
Variation in precipitation (**a**) and temperature (**b**) in the grasslands of Qilian County during the 2000–2019 period.

**Figure 10:**
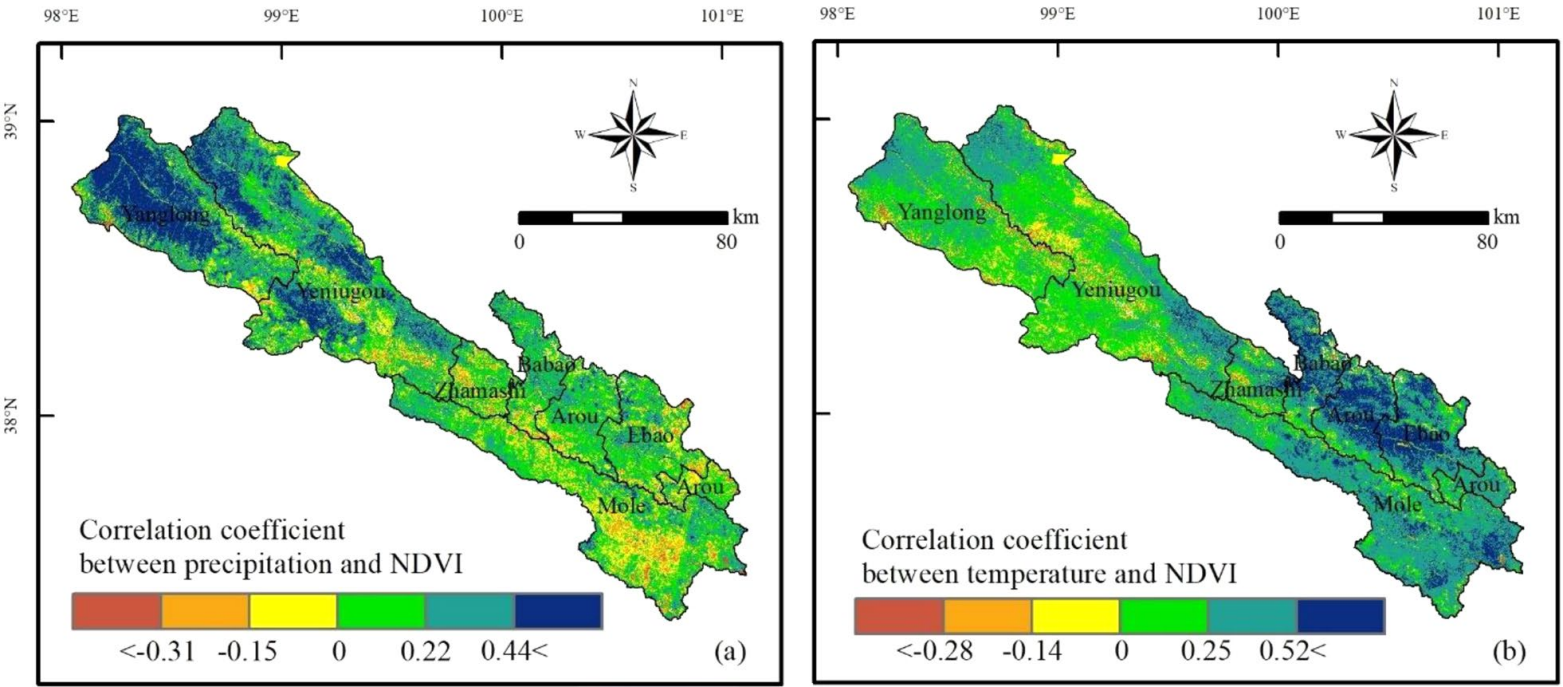
Correlation coefficients between NDVI and precipitation (**a**) and temperature (**b**). The map was generated by ArcGIS 10.2, URL: https://www.esri.com.

**Figure 11:**
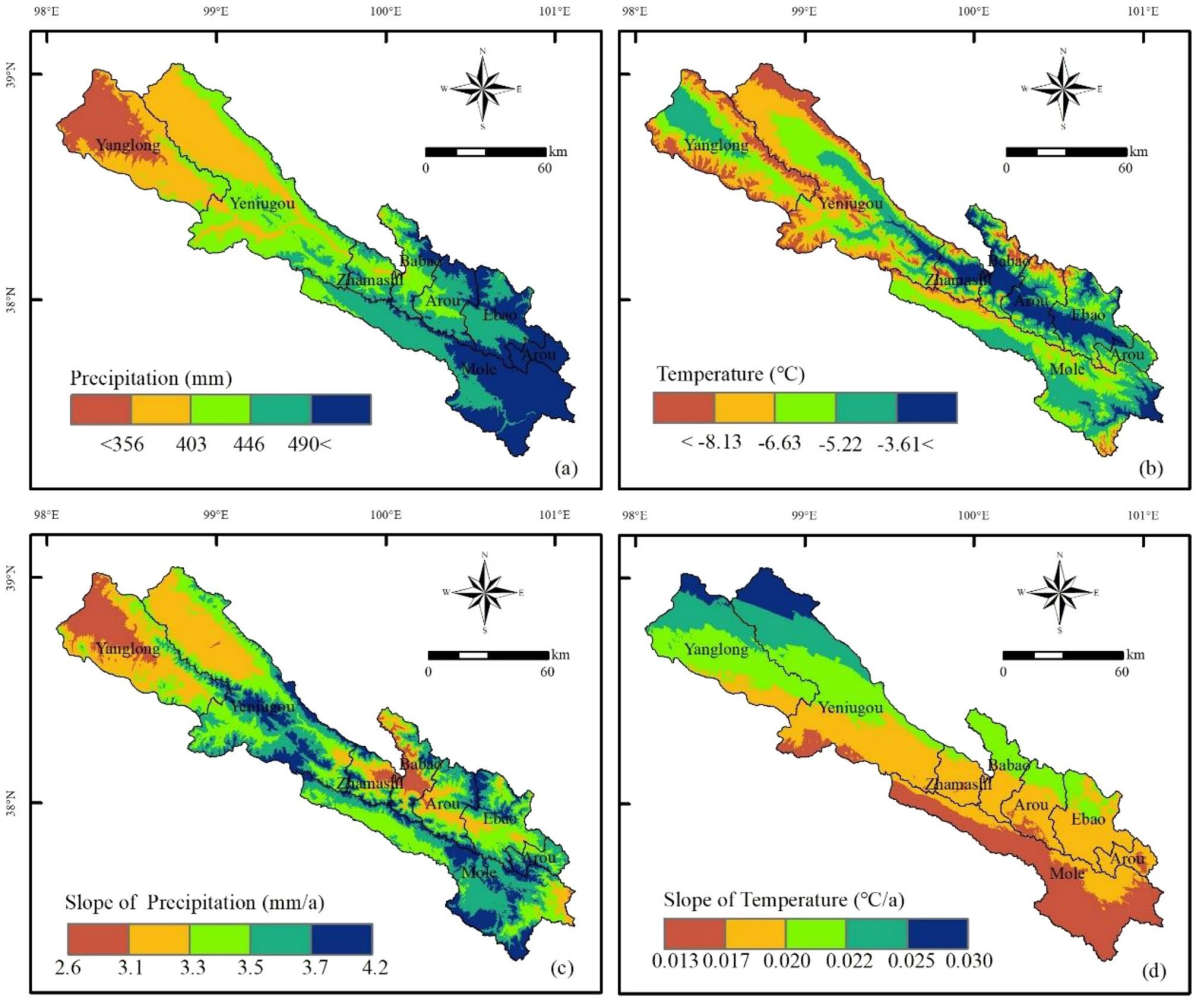
Spatial distribution (**a**,**b**) and variation (slopes) (**c**,**d**) in precipitation and temperature. The map was generated by ArcGIS 10.2, URL: https://www.esri.com.

In the 20 years study period, the temperature in Qilian County significantly increases (slope = 0.03; *P* < 0.05), while precipitation shows an insignificant increasing trend (Fig. 9). Water is generally regarded as the most critical ingredient influencing plant growth, hence water shortage may have a significant impact on vegetation production, especially in arid and semi-arid regions^3,57,58^. Temperature is also an indirect indicator of the available energy for vegetation growth, thus a rise in temperature could affect the greenness of the vegetation and severely limit the growth of shrubs and sparse vegetation^59^. Ji et al. discovered that change in forest NPP in China were mostly induced by changes in precipitation, followed by temperature^60^. Considering the geographical differences of climate change, we further computed the correlation coefficients between NDVI and temperature and precipitation on a pixel scale to study how climate change affects grassland ecosystems (Fig. 10). NDVI is positively correlated with temperature and precipitation in most areas. Specifically, the areas where NDVI show a positive correlation with precipitation are concentrated in the northwest of Qilian County (Fig. 10a). The precipitation in these areas is relatively scarce, and the annual precipitation is less than 400 mm (Fig. 11a). Therefore, moisture is very important to grass growth in the area, even when NDVI is negatively correlated with temperature (Fig. 10b). From the spatial variation in temperature, the rate of temperature rise is greater than 0.02 °C/a (Fig. 11d). The rise of temperature usually increases the evaporation of surface water, which can have adverse effects on grass growth^8^, affect grassland coverage, and lead to degradation, which also explains to some extent why grassland degradation regions are primarily scattered in the northwest. Our findings are consistent with those of Zhou et al.^2^, who found that a wetter environment is conducive to vegetation development, yet in areas with less precipitation, warming can cause drought, which has a negative impact on plant growth^61^. Wu et al. and Duan and Xiao found that warming was detected over more than three-quarters of the land surface of the Qinghai–Tibet Plateau, with warming as the most common driving factor for alpine grassland dynamics^54,62^.

Due to the decrease in groundwater evaporation and enhancement of soil water retention, the slightly lower temperature (Fig. 11b) and increasing precipitation (Fig. 11a) have improved the grassland growth in the southeast of Qilian County^63,64^. Therefore, the regions of vegetation with improving conditions were mainly concentrated in these areas, such as Arou Township, Ebao Town, and Mole Town. This is because the soil water content is the key element affecting net primary productivity^9^. Increased precipitation (Fig. 11c) could increase soil water content and benefit the growth of grassland. On the other hand, from the spatial distribution of grassland degradation (Fig. 3a), some areas in the southeast of Qilian County have undergone grassland degradation to varying degrees, such as Babao Town and Mole Town. The possible reason is that the increase in precipitation exceeds the demand of vegetation growth, and it decreased solar radiation and increased relative humidity, which inhibited grassland photosynthesis^65^.

The different responses of different degraded or restored grasslands to climate factors are also attributed to changes in the water and heat cycles^50,66^. In the process of grassland degradation, vegetation coverage decreased, the influence of vegetation on water evaporation weakened, the rainfall infiltration rate decreased and bare grassland increased^50^. Bare soil enables a large amount of water and heat to enter the atmosphere, resulting in the instability of a grassland ecosystem, which in turn leads to the aggravation of degradation and changes in air exchanges between water and heat^66^. In addition, due to the differences of vegetation community structure and soil properties in different degradation stages, changes in vegetation productivity, soil moisture and the nitrogen cycle exhibit different responses to climate factors^67–69^. Xu et al. found that surface transpiration and infiltration may cause the decrease of soil moisture on the surface of grassland in the slight and moderate degradation stage. Low vegetation coverage, low plant activity level and reduced transpiration may cause the decrease in soil moisture in severe and extreme degradation stages^70^.

Furthermore, grassland degradation accounts for the largest proportion of degraded area at elevations of 3500–4500 m between 2000 and 2019 (Fig. S2), reducing the NDVI while the spatial heterogeneity increases, which is consistent with a prior study^48^. Above 4500 m altitude, 81.08% of the grassland has improved, indicating an increase in vegetation covering and a decrease in spatial heterogeneity. A recent study also discovered higher vegetation cover in the Himalayas at high altitudes^71^.

### Anthropogenic impacts on grassland degradation or restoration

Human activities are the principal drivers of grassland degradation or restoration^12^. This study found that the contribution of climate-driven grassland restoration was smaller than that of human activities-driven. According to Zhou et al.^2^, human activities-caused grassland restoration accounts for 78.1% of the total grassland area in China, including Qinghai, southeast of Tibet, Tianshan Mountains. On the one hand, this is mainly due to the control of grazing capacity and the implementation of intervention measures that have a positive impact on the grasslands in Qilian County. Grazing is the main factor influencing grassland change^72^. Moderate grazing can help to maintain the biodiversity of the grassland ecosystems^73^. According to Fig. S3, the overload rate of Qilian County shows a decreasing trend between 1982 and 2017, implying that overgrazing has declined. Generally, reducing grazing may have a positive impact on the soil environment, such as reducing the compaction of the surface soil, thus promoting restoration^74^. Duan et al. also highlighted that overgrazing in the Qilian Mountains has been greatly alleviated^75^. In addition, grazing prohibition and exclusion, forage-livestock balance, and other policies on grasslands can avoid degradation and mitigate the damage. These may also explain why climate change was the primary driver of grassland degradation^12^. Wang et al. found that 28.6% of grassland restoration was attributed to grazing prohibition in the Qinghai–Tibet Plateau^4^. On the other hand, it may also be related to various ecological protection measures implemented by the government, such as protecting natural forests^76^, converting farmland to forests or grasslands^77^, and establishing the Qilian Mountain National Park Nature Reserve, which have had a positive influence on the vegetation^75^. Furthermore, considering the moderately degraded areas around Babao Town and Arou Township with point-distribution, this may be related to the overgrazing surrounding residential plots, resulting in a loss in vegetation cover^78^. Reduced vegetation cover fosters the invasion of pikas (*Ochotona curzoniae*), resulting in the formation of soil patches and grassland degradation^79^. Liu et al. found that overgrazing surrounding the resident plots could be the reason for the point-distribution characteristics of grassland degradation in the Qinghai–Tibet Plateau^36^.

From the dynamic change trend, 16.0% and 9.7% of desert grassland and alpine and sub-alpine meadow, respectively, would have been improved, while the other grassland types would have undergone continuous degradation or changed from improvement to degradation, indicating that the desert grassland and alpine and sub-alpine meadow with decreasing trends in human impacts, and climate change promoted grassland improvement. This shift in grassland types may be caused by differences in plant species combinations and functional properties^54^, while the increased precipitation may improve the vegetation coverage in desert and grassland areas^80^.

In summary, the grassland restoration or degradation were jointly affected by climate change and human activities in Qilian County between 2000 and 2019. Different types of degraded grassland should implement different restoration strategies. Specifically, grassland degradation caused by climate change is difficult to control, because it is the normal response of grassland to environmental change^7^. The climate-driven grassland with slight degradation or re-growing (CDSR) regions were observed around Yanglong Township, the northwest of Yeniugou Township, and the north of Arou Town, owing to increasing warmth and decreased radiation. On the one hand, native grasses can be genetically modified to adapt to the changing climates, and aridity- and cold-resistant grasses should be introduced in these areas to mitigate the impact of unfavorable climate changes on grasslands^6,81^. In addition, irrigation is commonly employed to fulfill the water needs of plants and to ensure grass production^82^. On the other hand, the government should also establish a series of appropriate development models of tree-shrub-grasses configuration to further minimize grassland degradation induced by climate change^10^. In severely degraded regions, grassland biodiversity should be preserved through alternative measures such as rotational grazing and seasonal fencing^81^.

### Uncertainties and limitations

The residual trend analysis (RESTREND) has some drawbacks and limitations in assessing the impact of climate change and human activities on grassland dynamics. In the current study, the stepwise regression analysis and random forest regression models were used to select the dominant climate factors significantly affecting the NDVI to fit the regression equation with climate variables. However, this study only considered the impact of precipitation, temperature, sunshine hours, solar radiation, and wind speed on the NDVI, and did not consider other climate factors such as drought and extreme climate. However, this study could provide a reference method to reasonably select climate factors. In future research, a selection of the climate factors significantly related to NDVI in the study area as the climate variables for predicting NDVI is warranted and compare the applicability of different methods. And the cumulative and lagging effects of climate factors on NDVI will also be focused on in our future research. In addition, the variation in grassland was affected by grassland species, altitude, soil properties, grassland rodents, diseases and pests, and other factors in addition to climate factors and human activities^81^, which were not considered in this study because the quantitative methods for studying these factors at the macro scale are not mature.

In this study, we designed an evaluation method based on NDVI and CV_NDVI_, which distinguished the influence of climate change and human activities on different degraded grasslands, compensates for the shortcomings of previous studies, and is more convenient to evaluate the degree of grassland degradation caused by climate change and human activities in spatial terms. Nonetheless, this method also has some drawbacks. Although this study identified the driving factors of grassland degradation based on the relative roles of climate change and human activities on vegetation cover and spatial heterogeneity (ecosystem stability), the CV_NDVI_ calculation remains with great uncertainty. However, we provide a useful reference for distinguishing the impacts of climate change and human activities on different degraded grasslands. Moreover, this study aimed to assess the effects and relative roles of climate change and human activities on grassland degradation or restoration in Qilian County on a macro scale. Climate change and human activities need further study at a finer scale in combination with ground truth data.

## Conclusion

In the current study, NDVI and CV_NDVI_ were selected as indicators of grassland degradation and used the improve the commonly used residual trend analysis method to evaluate the driving factors of different degraded grasslands between 2000 and 2019; further, the Hurst index was used to analyze the forecasted change in degradation or restoration in Qilian County. The results showed that nearly 55.0% of the grasslands between 2000 and 2019 are degraded, mainly affected by slight degradation. Grassland restoration or degradation was jointly induced by climate change and human activities. The regions dominated by both factors presenting grassland improvement, slight degradation or re-growing, moderate degradation, and severe degradation or desertification accounted for 16.6%, 34.3%, 3.3%, and 1.3%, respectively, of the total grassland area. In addition, the area of climate-driven grassland improvement was less than that of human activities-driven (4.2% < 12.2%). Secondly, regardless of the degree of degradation, the grassland degradation area caused by climate change was larger than that caused by human activities, such as slight degradation or re-growing (12.3% vs. 9.5%), moderate degradation (2.3% vs. 1.5%), and severe degradation or desertification (1.2% vs. 1.1%).

Nearly 95.69% of the grassland in Qilian County will continue to degrade, including the previously degraded areas. To overcome some of the limitations of this study, future research should improve the evaluation methods, combined with ground truth data, identify the driving factors of different degraded grasslands on a finer scale, and predict the trend of grassland degradation or restoration. Our findings will not only help us understand the drivers of different degraded grasslands but also help decision-makers formulate grassland ecological management policies and guidelines.

## Supplementary Information

Supplementary Information.

## Data Availability

The datasets used and/or analysed during the current study available from the corresponding author on reasonable request.

## References

[1] Bi X (2020). Response of grassland productivity to climate change and anthropogenic activities in arid regions of Central Asia. Peer J..

[2] Zhou W (2017). Grassland degradation remote sensing monitoring and driving factors quantitative assessment in China from 1982 to 2010. Ecol. Indic..

[3] Liu YY (2019). Assessing the effects of climate variation and human activities on grassland degradation and restoration across the globe. Ecol. Indic..

[4] Zhang Y (2016). Vegetation dynamics and its driving forces from climate change and human activities in the Three-River Source Region, China from 1982 to 2012. Sci. Total Environ..

[5] Wang Z (2016). Quantitative assess the driving forces on the grassland degradation in the Qinghai-Tibet Plateau, China. Ecol. Inf..

[6] He CY, Tian J, Gao B, Zhao YY (2015). Differentiating climate- and human-induced drivers of grassland degradation in the Liao River Basin, China. Environ. Monit. Assess..

[7] Liu YY (2019). Grassland dynamics in responses to climate variation and human activities in China from 2000 to 2013. Sci. Total Environ..

[8] Jiang LL, Jiapaer G, Bao AM, Guo H, Ndayisaba F (2017). Vegetation dynamics and responses to climate change and human activities in Central Asia. Sci. Total Environ..

[9] Chen T (2019). Disentangling the relative impacts of climate change and human activities on arid and semiarid grasslands in Central Asia during 1982–2015. Sci. Total Environ..

[10] Gang C, Zhao W, Zhang Y, Zhao T, Gao X, Wen Z (2018). The impacts of land conversion and management measures on the grassland net primary productivity over the Loess Plateau, Northern China. Sci. Total Environ..

[11] Guo D, Wang H (2013). Simulation of permafrost and seasonally frozen ground conditions on the Tibetan Plateau, 1981–2010. J. Geophys. Res. Atmos..

[12] Yang Y (2016). Comparative assessment of grassland degradation dynamics in response to climate variation and human activities in China, Mongolia, Pakistan and Uzbekistan from 2000 to 2013. J. Arid Environ..

[13] Li CX, Jong R, Schmid B, Wulf H, Michael ES (2020). Changes in grassland cover and in its spatial heterogeneity indicate degradation on the Qinghai-Tibetan Plateau. Ecol. Indic..

[14] Li F, Chen W, Zeng Y, Zhao QJ, Wu BF (2014). Improving estimates of grassland fractional vegetation cover based on a pixel dichotomy model: A case study in Inner Mongolia, China. Remote Sens..

[15] Wang J, Brown DG, Chen JQ (2014). Drivers of the dynamics in net primary productivity across ecological zones on the Mongolian plateau. Landsc. Ecol..

[16] Han DM, Wang GQ, Xue BL, Liu TX, Yinglan LA, Xu XY (2018). Evaluation of semiarid grassland degradation in north China from multiple perspectives. Ecol. Eng..

[17] Liu HX, Zhang AB, Jiang T, Zhao AZ, Zhao YL, Wang D (2018). Response of vegetation productivity to climate change and human activities in the Shaanxi–Gansu–Ningxia region, China. J. Indian Soc. Remote Sens..

[18] Zheng K (2019). Impacts of climate change and human activities on grassland vegetation variation in the Chinese Loess Plateau. Sci. Total Environ..

[19] Yan YC, Liu XP, Wen YY, Ou JP (2019). Quantitative analysis of the contributions of climatic and human factors to grassland productivity in northern China. Ecol. Indic..

[20] Wang H (2016). Impacts of climate change on net primary productivity in arid and semiarid regions of China. Chin. Geogra. Sci..

[21] Thomas M (2014). Human land-use practices lead to global long-term increases in photosynthetic capacity. Remote Sens..

[22] Becerril-Pina R, Mastachi-Loza CA, Gonzalez-Sosa E, Diaz-Delgado C, Ba KM (2015). Assessing desertification risk in the semi-arid highlands of central Mexico. J. Arid Environ..

[23] Evans J, Geerken R (2004). Discrimination between climate and human-induced dryland degradation. J. Arid Environ..

[24] Meng M (2019). Vegetation change in response to climate factors and human activities on the Mongolian Plateau. Peer J..

[25] Burrell AL, Evans JP, Liu Y (2017). Detecting dryland degradation using time series segmentation and residual trend analysis (TSS-RESTREND). Remote Sens Environ..

[26] Gedefaw MG, Geli HME, Abera TA (2021). Assessment of rangeland degradation in New Mexico using time series segmentation and residual trend analysis (TSS-RESTREND). Remote Sens..

[27] Zhang F (2021). Changes of Grassland Net Primary Productivity in the Qinghai Tibet Plateau During the Past 34 Years and Analysis of Its Local Degradation Characteristics.

[28] Li LH (2018). Current challenges in distinguishing climatic and anthropogenic contributions to alpine grassland variation on the Tibetan Plateau. Ecol. Evol..

[29] Zhu ZC (2016). Greening of the earth and its drivers. Nat. Clim. Change..

[30] Song LC, Ma WW, Li G, Liu SN, Lu G (2021). Effect of temperature changes on nitrogen mineralization in soils with different degradation gradients in Gahai Wetland. Acta Pratacul. Sin..

[31] Dai LC (2021). Effect of grazing management strategies on alpine grassland on the northeastern Qinghai-Tibet Plateau. Ecol. Eng..

[32] Liu YY (2021). Evaluating the dynamics of grassland net primary productivity in response to climate change in China. Glob. Ecol. Conserv..

[33] Bestelmeyer BT, Duniway MC, James DK, Burkett LM, Havstad KM (2013). A test of critical thresholds and their indicators in a desertification-prone ecosystem: More resilience than we thought. Ecol. Lett..

[34] Kéfi S (2014). Early warning signals of ecological transitions: Methods for spatial patterns. PLoS ONE.

[35] Li JZ (2016). IKONOS image-based extraction of the distribution area of *Stellera*
*chamaejasme* L. in Qilian County of Qinghai Province, China. Remote Sens..

[36] Liu YQ, Lu CH (2021). Quantifying grass coverage trends to identify the hot plots of grassland degradation in the Tibetan Plateau during 2000–2019. Int. J. Environ. Res. Public Health..

[37] Kendall MG (1948). Rank Correlation Methods.

[38] Mann HB (1945). Nonparametric tests against trend. Econometrica.

[39] Zhang ZM, Lu CH (2020). Clustering analysis of soybean production to understand its spatiotemporal dynamics in the North China Plain. Sustainability..

[40] Pei TT (2021). The sensitivity of vegetation phenology to extreme climate indices in the Loess Plateau, China. Sustainability..

[41] Lu BB, Charlton M, Harris P, Fotheringham AS (2014). Geographically weighted regression with a non-Euclidean distance metric: A case study using hedonic house price data. Int. J. Geogr. Inf. Sci..

[42] Sun LQ, Zhang FH, Yang SW, Qiu AG, Zhang XL (2019). The method of selecting geographically and temporally weight regression variable based on stepwise regression. Sci. Surv. Mapp..

[43] Jiang WG, Yuan LH, Wang WJ, Cao R, Zhang YF, Shen WM (2015). Spatio-temporal analysis of vegetation variation in the Yellow River basin. Ecol. Indic..

[44] Ndayisaba F, Guo H, Bao A, Guo H, Karamage F, Kayiranga A (2016). Understanding the spatial temporal vegetation dynamics in Rwanda. Remote Sens..

[45] Kéfi S (2007). Spatial vegetation patterns and imminent desertification in Mediterranean arid ecosystems. Nature.

[46] Chen JJ, Yi SH, Qin Y (2017). The contribution of plateau pika disturbance and erosion on patchy alpine grassland soil on the Qinghai-Tibetan Plateau: Implications for grassland restoration. Geoderma.

[47] Cai HY, Yang XH, Xu XL (2015). Human-induced grassland degradation/restoration in the central Tibetan Plateau: The effects of ecological protection and restoration projects. Ecol. Eng..

[48] Wang P, Lassoie JP, Morreale SJ, Dong SK (2015). A critical review of socioeconomic and natural factors in ecological degradation on the Qinghai-Tibetan Plateau. China. Rangel. J..

[49] Lu CB, Hou LF (2018). Cause analysis and Control Countermeasures of grassland degradation in Qilian County, Qinghai Province. Today Anim. Husb. Vet. Med..

[50] Guo XW (2020). Light grazing significantly reduces soil water storage in Alpine Grasslands on the Qinghai-Tibet Plateau. Sustainability..

[51] Bai YF (2020). Climate warming benefits alpine vegetation growth in Three-River Headwater Region, China. Sci. Total Environ..

[52] Chen T (2020). Unraveling the relative impacts of climate change and human activities on grassland productivity in Central Asia over last three decades. Sci. Total Environ..

[53] Li A, Wu JG, Huang JH (2012). Distinguishing between human-induced and climate-driven vegetation changes: A critical application of RESTREND in inner Mongolia. Landsc. Ecol..

[54] Wu JS (2020). Disentangling climatic and anthropogenic contributions to nonlinear dynamics of alpine grassland productivity on the Qinghai-Tibetan Plateau. J. Environ. Manag..

[55] Gang C (2015). Comparative assessment of grassland NPP dynamics in response to climate change in China, North America, Europe and Australia from 1981 to 2010. J. Agron. Crop Sci..

[56] Gang CC (2014). Quantitative assessment of the contributions of climate change and human activities on global grassland degradation. Environ. Earth Sci..

[57] Chen YZ (2016). Grassland carbon sequestration ability in China: A new perspective from terrestrial aridity zones. Rangeland Ecol. Manag..

[58] Mowll W (2015). Climatic controls of aboveground net primary production in semi-arid grasslands along a latitudinal gradient portend low sensitivity to warming. Oecologia.

[59] Zhou Y (2015). Climate contributions to vegetation variations in central Asian Drylands: Pre- and post-USSR collapse. Remote Sens..

[60] Ji Y (2020). Variation of net primary productivity and its drivers in China’s forests during 2000–2018. For. Ecosyst..

[61] Zeng B, Yang TB (2008). Impacts of climate warming on vegetation in Qaidam Area from 1990 to 2003. Environ. Monit. Assess..

[62] Duan AM, Xiao ZX (2015). Does the climate warming hiatus exist over the Tibetan Plateau?. Sci. Rep..

[63] Fang JY (2005). Precipitation patterns alter growth of temperate vegetation. Geophys. Res. Lett..

[64] Zhao X, Tan K, Zhao S, Fang J (2011). Changing climate affects vegetation growth in the arid region of the northwestern China. J. Arid Environ..

[65] Ukkola AM (2016). Reduced streamflow in water-stressed climates consistent with CO_2_ effects on vegetation. Nat. Clim. Change..

[66] Dong SK, Shang ZH, Gao JX, Boone RB (2019). Enhancing sustainability of grassland ecosystems through ecological restoration and grazing management in an era of climate change on Qinghai-Tibetan Plateau. Agric. Ecosyst. Environ..

[67] Xu HP (2019). Responses of plant productivity and soil nutrient concentrations to different alpine grassland degradation levels. Environ Monit Assess..

[68] Wen WY (2016). Research on soil net nitrogen mineralization in Stipa grandis grassland with different stages of degradation. Geosci J..

[69] She Y (2022). Vegetation attributes and soil properties of alpine grassland in different degradation stages on the Qinghai-Tibet Plateau, China: A meta-analysis. Arab J Geosci..

[70] Xu GP (2006). Study on the Change of Vegetation and Soil Nutrients of Alpine Meadow Under Different Degradation Degrees in Eastern Qilian Mountains.

[71] Anderson K (2020). Vegetation expansion in the subnival Hindu Kush Himalaya. Glob. Chang. Biol..

[72] Chen BX (2014). The impact of climate change and anthropogenic activities on alpine grassland over the Qinghai-Tibet Plateau. Agric. For. Meteorol..

[73] Zhang XW, Li G, Dong KH, Zhao X (2019). Effects of grazing and enclosure on community characteristics and biodiversity in Leymus chinensis grassland. J. Grassl. Forage Sci..

[74] Huang K (2016). The influences of climate change and human activities on vegetation dynamics in the Qinghai-Tibet Plateau. Remote Sens..

[75] Duan QT, Luo LH, Zhao WZ, Zhuang YL, Liu F (2021). Mapping and evaluating human pressure changes in the Qilian mountains. Remote Sens..

[76] Wang Y (2020). Performance and obstacle tracking to natural forest resource protection project: A rangers’ case of Qilian mountain, China. Int. J. Environ. Res. Public Health..

[77] Li ZY (2020). Changes in nutrient balance, environmental effects, and green development after returning farmland to forests: A case study in Ningxia, China. Sci. Total Environ..

[78] Li CX, de Jong R, Schmid B, Wulf H, Schaepman ME (2019). Spatial variation of human influences on grassland biomass on the Qinghai-Tibetan plateau. Sci. Total Environ..

[79] Li XL (2011). Rangeland degradation on the Qinghai-Tibet Plateau: Implications for rehabilitation. Land Degrad. Dev..

[80] Li CB (2014). Regional vegetation dynamics and its response to climate change—a case study in the Tao River Basin in Northwestern China. Environ. Res. Lett..

[81] Liu YY (2021). Untangling the effects of management measures, climate and land use cover change on grassland dynamics in the Qinghai-Tibet Plateau, China. Land Degrad. Dev..

[82] Hou X (2013). Chinese Grassland Science.

