# Supplementary material for: Impacts of climate change and human activities on different degraded grassland based on NDVI
Source: Sci Rep. 2022 Sep 23;12:15918. doi: 10.1038/s41598-022-19943-6 (PMC9508234; doi:10.1038/s41598-022-19943-6)
Supplement: Supplementary file 1 — Supplementary Information. [file 41598_2022_19943_MOESM1_ESM.docx]

**Table S1**. NDVI stepwise regression results

| Variables | RMSE | R-square |
| --- | --- | --- |
| x4 | 0.1563 | 0.4583 |
| x2, x4 | 0.1551 | 0.4675 |
| x2, x3, x4 | 0.1544 | 0.4733 |
| x1, x2, x3, x4 | 0.1543 | 0.4746 |
| x1, x2, x3, x4, x5 | 0.1545 | 0.4748 |
| x1, x2, x3 | 0.1543 | 0.4740 |

Note: RMSE: Standard error;

x1: Precipitation; x2: Temperature; x3: Sunshine hours; x4: Wind speed; x5: Solar radiation;

**Table S2**. Multiple linear regression results of different variables

| Variables | R^2^ | P |
| --- | --- | --- |
| Precipitation and Temperature | 0.4116 | 0.01 |
| Precipitation, Temperature and Sunshine hours | 0.4815 | 0.01 |


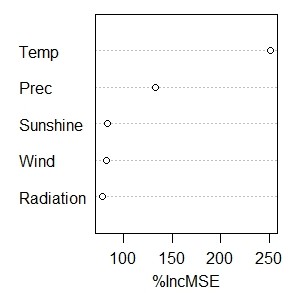


Fig. S1. Climatic factors affecting the normalized difference vegetation index (NDVI) and their influence degree


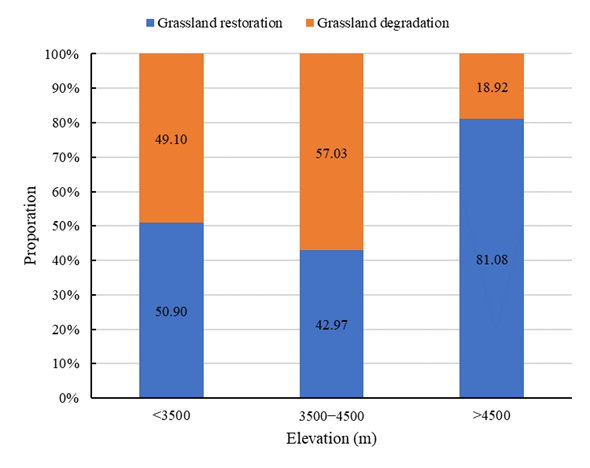


Fig. S2. Proportion of grassland degradation or restoration along altitude gradients


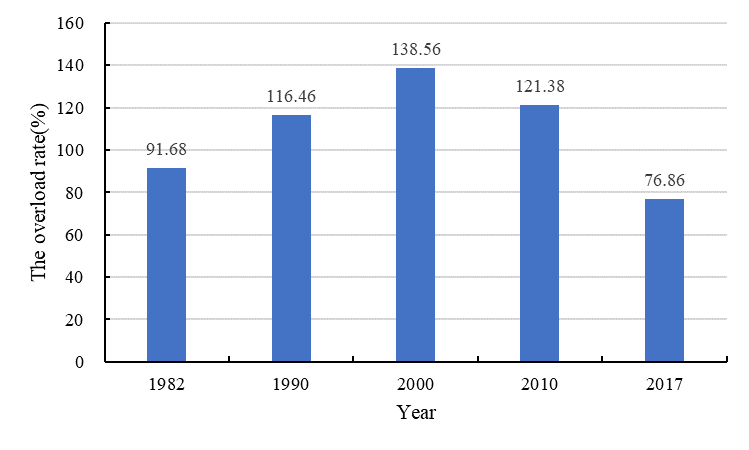


Fig. S3. Proportion of grassland degradation or restoration along altitude gradients
